# Psychiatric Symptoms Associated with Non-Celiac Gluten Sensitivity: A Focused Narrative Review

**DOI:** 10.3390/nu18142272

**Published:** 2026-07-11

**Authors:** Dragica Pavlovic, Marina Gazdic Jankovic, Dragana Papic, Nikolina Kastratovic, Nikola Jovic, Simona Protrka, Vladimir Janjic, Biljana Ljujic

**Affiliations:** 1 Department of Genetics, Faculty of Medical Sciences, University of Kragujevac, 69 Svetozar Markovic Street, 34000 Kragujevac, Serbia; dragica.miloradovic8@gmail.com (D.P.); drmiloradovic7@gmail.com (D.P.); n_kastratovic@outlook.com (N.K.); bljujic74@gmail.com (B.L.); 2 Center for Harm Reduction of Biological and Chemical Hazards, Faculty of Medical Sciences, University of Kragujevac, 69 Svetozar Markovic Street, 34000 Kragujevac, Serbia; 3 Department of Gynecology and Obstetrics, Faculty of Medical Sciences, University of Kragujevac, 69 Svetozar Markovic Street, 34000 Kragujevac, Serbia; docctorny@gmail.com; 4 Clinic of Gynecology and Obstetrics, University Clinical Center Kragujevac, Zmaj Jovina 30, 34000 Kragujevac, Serbia; 5 Pediatric Clinic, University Clinical Centre Kragujevac, Zmaj Jovina 30, 34000 Kragujevac, Serbia; protrka.simona@gmail.com; 6 Department of Psychiatry, Faculty of Medical Sciences, University of Kragujevac, 69 Svetozar Markovic Street, 34000 Kragujevac, Serbia; vladadok@yahoo.com; 7 Psychiatry Clinic, University Clinical Center Kragujevac, Zmaj Jovina 30, 34000 Kragujevac, Serbia

**Keywords:** non-celiac gluten sensitivity, psychiatric symptoms, intestinal barrier, microbiota, gut dysbiosis, gut–brain axis

## Abstract

Non-celiac gluten sensitivity (NCGS), even though it is a term defined for more than four decades, is still an enigmatic clinical entity. This is primarily due to the absence of specific diagnostic criteria, with most reports relying on patients’ self-diagnosis. Individuals with NCGS experience intestinal (abdominal pain, bloating, diarrhea, or constipation) and extraintestinal symptoms (“foggy mind,” depression, anxiety, and deterioration of psychiatric conditions) after gluten consumption. Frequent disregard and oversight of extraintestinal manifestations, such as psychiatric symptoms, further complicates NCGS. The lack of understanding about NCGS causes individuals living with this condition to experience distress, frustration, and stigmatization. An impaired intestinal barrier, gut dysbiosis, and alterations in the gut–brain axis are proposed as key factors in the NCGS and psychiatric symptoms associated with NCGS. None of these links are yet definitive, and the methodological limitations of existing studies—small samples, self-reported diagnoses, and insufficient blinding—demand that conclusions be drawn with appropriate restraint. The purpose of this review is to provide an up-to-date understanding of the pathophysiology of NCGS and psychiatric symptoms associated with NCGS. Future studies should focus on development of reliable, objective diagnostic markers for NCGS and on the design of adequately powered, double-blind, placebo-controlled trials that incorporate validated psychiatric outcome measures alongside intestinal indices.

## 1. Introduction

“Gluten-related disorders (GRDs)” encompass various immune-mediated conditions associated with gluten consumption, primarily including celiac disease (CD), wheat allergy (WA), and non-celiac gluten sensitivity (NCGS), also known as wheat sensitivity [1]. NCGS is defined by the onset of intestinal and/or extraintestinal symptoms following the intake of gluten-containing cereals [2]. The prevalence of NCGS is between 0.6% and 13% of the total population and it is elevated during the third to fourth decades of life among individuals with a history of autoimmune and functional gastrointestinal disorders [3,4]. Additionally, evidence of health benefits from a gluten-free diet (GFD) in individuals without CD has led to the recognition of NCGS as a distinct clinical disorder [5]. Specific serological indicators for NCGS are not well-defined, in contrast to CD; only a fraction of patients have increased antibodies to gluten peptides, and mucosal damage is generally absent [6]. However, in the absence of particular tests and biomarkers, the sole dependable approach for identifying individuals with NCGS is via an exclusion diagnosis [7], as outlined by the Salerno criteria [8]. The Salerno experts put forward a two-step diagnostic protocol for NCGS (Table 1). The first step is designed to establish whether a patient genuinely responds to a GFD: following a baseline period of at least six weeks on a normal, gluten-containing diet, patients transition to a GFD and are monitored weekly for a further six weeks using a symptom questionnaire scored on a 1–10 numerical rating scale [5]. A patient is classified as a responder if their nominated symptom score decreases by at least 30% in at least three of these six weekly evaluations. The second step, which serves to confirm the diagnosis, consists of a double-blind, placebo-controlled gluten challenge: after at least four weeks of strict adherence to a GFD, patients undergo a one-week gluten challenge (approximately 8 g/day, cooked and homogeneously distributed within the food vehicle) and a one-week placebo challenge, administered in cross-over order and separated by a one-week washout period on strict GFD. A positive result requires a variation of at least 30% in the main symptom score between the gluten and placebo arms [5]. Table 1 summarizes these elements: the six-week supervised GFD trial with its ≥30% responder criterion, the blinded gluten-versus-placebo re-challenge conducted over one week per arm with an interposed washout period and approximately 8 g of gluten per day, and the response threshold ultimately used to confirm the diagnosis.

In 1956, a case series indicated a potential association between gluten and mood as well as psychiatric symptoms in individuals without CD [9]. Similarly, recent studies have indicated that mood symptoms are often associated with wheat consumption [10], with ‘low mood’ frequently cited as a reason for avoiding gluten [11], even in individuals without CD or WA. On top of that, current clinical investigations have identified elevated gluten-related antibodies in patients with bipolar disorder, major depressive disorder, and schizophrenia [12,13,14], while episodes of acute mania may correlate with increased serum levels of antibodies against gliadin [15]. Consequently, there is increasing evidence for a potentially bidirectional relationship between gluten sensitivity and psychiatric disorders. But still, NCGS continues to be a subject of scientific debate, resulting in a lack of clarity for patients and society regarding gluten sensitivity beyond CD [16]. Prior research indicated the participation of the immune system, intestinal inflammation, dysbiosis, impaired intestinal barrier and gut–brain axis dysregulation in the pathophysiology of NCGS [17]. Given that these debilitating conditions impose significant physical and emotional costs on affected individuals and their families, as well as a significant financial burden on society, there has been an increased focus on identifying the key mechanisms involved in the development of gluten-related psychiatric disorders. In this review, we address the present understanding of pathophysiology of NCGS and provide an up-to-date synthesis of the psychiatric symptoms associated with NCGS.

## 2. Materials and Methods

### Literature Search Strategy

This focused narrative review examined the available evidence on psychiatric symptoms associated with non-celiac gluten sensitivity (NCGS), with particular attention to depression, anxiety, psychosis/schizophrenia, as well as to the principal mechanisms proposed to underline these manifestations, including intestinal dysbiosis, epithelial barrier dysfunction, immune activation, and gut–brain axis alterations. Because interpretation of this field is complicated by substantial overlap with other conditions, the search also included studies addressing fermentable oligo-, di-, monosaccharides and polyols (FODMAPs), irritable bowel syndrome (IBS), nocebo effects, and the nutritional consequences of a gluten-free diet.

A structured search of PubMed/MEDLINE, Scopus, Web of Science, and APA PsycINFO was performed from database inception to 14 April 2026 using combinations of free-text terms related to NCGS and psychiatric manifestations. The main search terms included “non-celiac gluten sensitivity”, “NCGS”, “non-celiac wheat sensitivity”, “NCWS”, “gluten sensitivity”, “wheat sensitivity”, “psychiatric”, “neuropsychiatric”, “mental health”, “depression”, “anxiety”, “mood”, “psychosis”, “schizophrenia”, “gut–brain axis”, “microbiota”, “dysbiosis”, “intestinal permeability”, “zonulin”, “immune response”, “inflammation”, “gliadin”, “short-chain fatty acids”, “lipopolysaccharide”, “FODMAP”, “IBS”, “nocebo”, and “gluten-free diet”.

Original clinical studies, observational studies, randomized studies, double-blind placebo-controlled gluten-challenge studies, case reports, case series, systematic reviews, meta-analyses, and selected preclinical studies were considered. The number of systematic reviews and meta-analyses (4), narrative/expert reviews (41), randomized controlled trials, including double-blind placebo-controlled gluten-challenge trials (8), other original clinical and observational studies (cross-sectional, cohort, case–control) (63), case reports (4) and case series (4), Animal (in vivo) and in vitro/preclinical studies (18). Of the 144 references cited, 63 (43.8%) were published within the last 10 years (2016–2026), reflecting the rapidly evolving understanding of NCGS, while a smaller set of foundational studies —including Dohan’s early schizophrenia–gluten investigations [18,19,20,21], the original 1956 case series describing wheat sensitivity and mood, and classic tryptophan-depletion studies from the 1980s–1990s—were retained for their conceptual and historical significance in establishing the gluten–psychiatric symptom link. Priority was given to human studies involving suspected or confirmed NCGS, particularly those using diagnostic approaches consistent with the Salerno criteria or double-blind placebo-controlled gluten challenge. Studies focused exclusively on celiac disease or wheat allergy were excluded unless they provided important comparative or theoretical context relevant to NCGS. Reference lists of relevant articles and review papers were also screened manually to identify additional studies.

## 3. Pathophysiology of NCGS and Associated Psychiatric Symptoms

**Figure 1:**
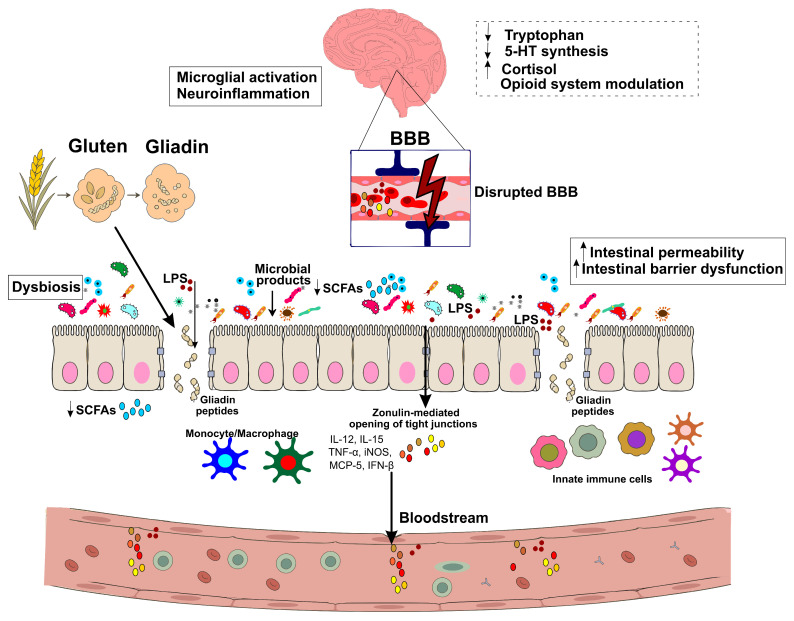
Schematic figure of potential pathophysiological mechanisms of psychiatric symptoms associated with NCGS. Intestinal dysbiosis, intestinal epithelial dysfunction, and increased intestinal permeability consequently leading to passage of gliadin, innate immune response-induced inflammatory process and impaired blood–brain barrier are crucial factors in pathophysiology of psychiatric symptoms associated with NCGS. Abbreviations: NCGS, non-celiac gluten sensitivity; BBB, blood–brain barrier; LPS, lipopolysaccharide; SCFA, short-chain fatty acid; 5-HT, 5-hydroxytryptamine (serotonin); IL-12, interleukin-12; IL-15, interleukin-15; TNF-α, tumor necrosis factor alpha; iNOS, inducible nitric oxide synthase; MCP-5, monocyte chemoattractant protein 5; IFN-β, interferon beta.

The pathophysiology of NCGS is not well understood; however, several studies have identified immune response, gut dysbiosis, and impaired intestinal epithelial barrier as significant factors in the development of NCGS (Table 2 and Figure 1) [22,23,24,25,26].

### 3.1. Intestinal Immune Response, Dysbiosis, and Impaired Intestinal Epithelial Barrier Dysfunction

Gluten is a type of prolamin that is mostly found in wheat, barley, rye, and oats. It is composed of gliadin and glutenin proteins. Dietary gluten has been linked to a harmful immune response in predisposed individuals [27,28,29,30]. After gluten is ingested, proteases in the digestive system break it down into peptides that are around 10 to 30 amino acids long. These peptides then cross the intestinal barrier through transcellular or paracellular transport. Intestinal tissue transglutaminase-2 (tTG2) deamidates these peptides, which makes them more susceptible to binding to major histocompatibility complex II (MHC II) molecules and initiating an inflammatory response. Specifically, in patients with NCGS, who are negative for DQ2 and DQ8, it is postulated that gluten proteins, together with other wheat constituents (wheat germ agglutinin and amylase–trypsin inhibitors), provoke an innate immune response [27,28,29,30]. Consequently, due to a diminished tolerance to gluten proteins, patients with CD and NCGS endure gastrointestinal irritation, exhaustion, and pain, characterized by inflammation and heightened permeability of the intestinal mucosa [31]. Studies indicate that gluten or wheat consumption leads to changes in the expression of innate immune components in individuals sensitive to wheat (Figure 1). These alterations include mucosal Toll-like receptor-2 (TLR-2), interleukin-10 (IL-10) derived from peripheral blood mononuclear cells, granulocyte colony-stimulating factor (GCSF), transforming growth factor-α (TGF-α), and chemokine CXCL-10 [24]. A diminished expression of forkhead box P3 (FOXP3) has been observed in intestinal biopsies from patients with NCGS compared to healthy individuals and those with CD [32,33,34,35]. Increased levels of interferon-γ (IFN-γ) have been observed in patients with NCGS following gluten exposure [36]. In addition to the role of the innate immune system, evidence indicated the involvement of the adaptive immune system in NCGS, as approximately 50% of NCGS patients exhibited elevated levels of anti-gliadin antibodies (AGAs) [37]. However, the absence of specific markers, such as anti-TG2 antibodies and anti-deamidated gliadin antibodies, suggests a mechanism distinct from that of CD. Some studies have documented intestinal inflammation in patients with NCGS, characterized by elevated eosinophil levels, increased intraepithelial CD3+T cells, and heightened lamina propria CD45+ cells [38].

The gut microbiota constitutes an essential element of the gut ecosystem, significantly influencing human health. It contributes to the development and maturation of the immune system, serves as a barrier against pathogens, aids in the absorption of nutrients and medications, and regulates metabolic intake [39]. An imbalance in the gut microbiota can result in a range of gastrointestinal and extra intestinal disorders [40]. Patients with NCGS exhibit a distinct microbial profile, characterized by a significant reduction in microbial richness, which signifies a less diversified gut microbiota [41]. NCGS is marked by particular alterations at the phylum level, notably an increase in *Ruminococcaceae* and a decrease in *Bacteroidetes* and *Fusobacteria*, illustrating a unique microbial community [42,43]. Multiple structures and mechanisms inhibit the translocation of microbes from the intestinal lumen into host tissues, notably the mucous layer (via the secretion of antimicrobial peptides), the intestinal epithelial barrier (IEB), and tight junctions. Thus, intestinal dysbiosis appears to play a role in epithelial barrier dysfunction and the related inflammatory response to gluten, thus contributing to the pathogenesis of NCGS (Figure 1) [42].

The dysregulation of the zonulin pathway can enhance the permeability of tight junctions, resulting in heightened vulnerability to inflammation and autoimmune diseases, as noted in CD [31]. Elevated zonulin levels have been observed in the serum of individuals with NCGS [44]. Research has proposed measuring the serum level of this protein as a biomarker for NCGS diagnosing and distinguishing it from IBS-D. Zonulin levels exhibited a correlation with common symptoms in patients with NCGS, including abdominal distension and pain [44]. Gut bacteria contribute to immune tolerance of gluten by producing short chain fatty acids (SCFAs). SCFAs contribute to the maintenance of the epithelial barrier via tight junctions, and play a role in epithelial growth and regeneration, the production of T-reg cells, and the pathogenic response to bacteria [45,46,47,48,49,50]. NCGS has been associated with reduced fecal acetate levels compared to healthy controls, suggesting a potential negative influence of this condition on SCFA production [45]. Concentrations of lipopolysaccharide (LPS)-binding proteins in sera and antibody reactivity to microbial products were increased in patients with NCGS. These findings correlate with circulating levels of intestinal fatty-acid-binding protein, implying a compromised gut barrier and microbial translocation [25,51]. Thus, imbalanced gut microbiota contributes to barrier dysfunction and interacts with the innate and adaptive immune system [52]. Increased intestinal permeability facilitates the entry of gliadin and other macromolecular antigens into the lamina propria, prompting the host to activate first-line defense mechanisms, including neutrophil recruitment to the exposure site (Figure 1) [53]. The inflammatory effects of gluten may extend beyond the gastrointestinal system. Elevated intestinal permeability facilitates the entry of toxic digestion byproducts, bacteria, and bacterial toxins into the bloodstream, potentially allowing them to access the central nervous system (CNS) [31]. In that sense, this condition encompasses many patients formerly classified as having “irritable bowel syndrome” or “psychosomatic disorder” [54]. In the absence of CD, NCGS patients have indicators of systemic immune activation, inflammation, and gut epithelial cell destruction, providing a biological basis for understanding gluten-related intestinal and extra-intestinal symptoms (schematic figure summarizing potential pathophysiological mechanisms of NCGS-induced psychiatric symptoms is provided in Figure 1).

### 3.2. Gut–Brain Axis Dysregulation and Neuropsychiatric Mechanisms

The pathophysiology of NCGS is still inadequately comprehended, with evidence coming from human, animal, and in vitro studies suggesting multiple potential mechanisms—none yet definitive (biological pathways implicated in NCGS-related psychiatric symptoms and strength of supporting data are summarized in Table 2) [55,56,57].

A complex and multifaceted relationship exists between mood and gluten-related disorders. The gut–brain axis is recognized as a bidirectional pathway that encompasses neural, endocrine, immune, and humoral networks influenced by the gut microbiome, with emerging evidence suggesting its role in influencing mental health regulation [58,59,60]. In NCGS, several authors have suggested that an altered gut–brain axis may help explain the neuropsychiatric symptoms reported by some patients [60,61], but the underlying human data are still relatively limited, and better designed prospective research is necessary before firm causal assumptions may be inferred (Table 2) [56,61].

Various theories concerning the etiology of mood symptoms in individuals with gluten-related disorders have been proposed. One of the theories posited that an immune response to gluten could result in depressive symptoms [62]. Other than that, the consumption of FODMAPs (Fermentable Oligo-, Di-, Mono-saccharides And Polyols—short-chain carbohydrates found in wheat, rye, barley, beans, pulses, and certain vegetables) has been proposed to exacerbate both physical and psychological symptoms in individuals believed to be gluten-sensitive [42,63]. Particularly, it is important to recognize that patients with functional gastrointestinal disorders often exhibit anxiety and depression, especially as a personality trait, which may contribute to the development and/or perception of symptoms [64]. Short-term exposure to gluten specifically elicited current depressive feelings in patients with self-reported NCGS, without affecting other indices or emotional disposition [65]. The variability in the effect of gluten on gastrointestinal and potentially extra-intestinal symptoms may be attributed to numerous influential variables that contribute to psychological dysfunction, including both biological and psychosocial factors [66]. Alternative explanations for the association between gluten and depression include irregularities in serotonin production, changes in cortisol secretion, the impact of gluten exorphins on the central nervous system, and alterations in gut microbiota (Figure 1) [67,68,69]. Serotonergic dysfunction resulting from reduced availability of tryptophan has been implicated in several psychological conditions, including depression [70,71,72,73]. Also, the tryptophan–serotonin–depression axis is robustly supported by acute tryptophan depletion (ATD) and metabolic studies in humans and animals [74,75,76]. However, wheat/gluten-specific tryptophan and serotonin changes are only demonstrated in a small number of rodent protein-comparison studies [77,78]. The production of 5-HT in the brain relies on the availability of its amino acid precursor, tryptophan. Hence, one research article has established a connection between protein intake, tryptophan synthesis, and the levels of 5-HT in the brain [77]. This study indicated that rats fed food-grade wheat for 2 h experienced modest reductions in brain tryptophan levels, suggesting that the 5-HT pathways are notably responsive to different proteins found in food [77]. However, no study has yet shown that gluten ingestion in humans directly lowers brain tryptophan or 5-HT, and the proposed mechanism remains hypothetical, as acknowledged in clinical and review articles [65] (Table 2). Other than that, one possible explanation could be changes in cortisol secretion, as higher circulating concentrations of cortisol are associated with negative effect, including aversive moods such as anxiety, hostility, and depression [79]. It is currently unclear whether this link is caused by stable individual variations (traits) or transient differences in affect (states) [80]. Despite this, it must be noted that currently, there is no evidence to suggest that gluten ingestion stimulates cortisol secretion; however, this relationship has been infrequently investigated [81].

Numerous investigations have highlighted a possible link between gluten and psychosis [18,82,83,84,85,86,87,88,89,90,91,92,93,94,95,96,97,98,99], as well as other neuropsychiatric disorders [100,101,102,103]. However, this remains a disputed issue that requires thoroughly planned prospective research to determine if gluten really triggers these disorders [99,104]. Neuropsychiatric symptoms associated with gluten may result from the excessive absorption of opioid-active peptides generated from the incomplete breakdown of gluten. Increased intestinal permeability, known as “leaky gut syndrome,” may permit these peptides to traverse the intestinal membrane, enter the bloodstream, and penetrate the blood–brain barrier (BBB), thereby influencing the endogenous opiate system and neurotransmission within the nervous system (Table 2 and Figure 1) [104,105]. The overexpression of zonulin, also known as haptoglobin-2, may contribute to the disruption of the BBB in a manner analogous to its role in enhancing intestinal permeability [106,107]. The observation that zonulin analogues can modulate the BBB by enhancing its permeability to high molecular weight markers and chemotherapeutic agents supports this hypothesis [108]. On the contrary, two independent human in vivo studies argue against a meaningful role for zonulin at the BBB [106]. The first used the cerebrospinal fluid-to-serum albumin quotient (Q-albumin), a well-established index of blood–brain barrier integrity in which a higher ratio reflects greater barrier permeability and found no association with zonulin. The second used dynamic contrast-enhanced magnetic resonance imaging (DCE-MRI), an imaging technique that quantifies the leakage of an intravenous contrast agent across the barrier in vivo and likewise found that zonulin plays a negligible role in BBB permeability. Adding to that, gliadin peptides have been shown to trigger innate immune activation in human monocytes and macrophages in vitro [109,110]. In parallel, transglutaminase 6 (TG6) is a neuronal transglutaminase widely expressed in the CNS and identified as the autoantigen in gluten ataxia and other gluten-related neurological disorders, capable of deamidating gliadin and giving rise to TG6-directed autoimmunity [98,111]. Together with neuropathological evidence of microglial and macrophage activation in gluten-related neurological disease [112,113], these findings support the hypothesis that gluten-driven innate immune responses could lead to the exposure and targeting of neuronal TG6 within the brain. In addition, earlier work has shown that gliadin can provoke cytokine release from monocytes and macrophages [114]. When mouse peritoneal macrophages were treated with various concentrations of gliadin, a clear rise in the expression of several pro-inflammatory genes was observed—namely TNF-α, IL-12, IL-15, IFN-β, iNOS, IP-10, and MCP-5. Collectively, these findings indicate that gliadin and its active peptides broadly enhance the expression of inflammatory genes [115]. *Ashkenazi* et al. demonstrated that lymphocyte responses to stimulation with gluten subfractions in a subgroup of schizophrenic patients resembled those of lymphocytes from patients with CD [116]. Finally, altered permeability, changes in gut microbiota, and infections may represent other potential pathogenetic mechanisms associated with schizophrenia and mood disorders in patients with NCGS [117]. However, it remains unclear whether alterations primarily pertain to intestinal degradation, absorption, immune response to gliadin, autoantibody cross-reactivity, the production of neuroactive peptides due to altered digestion processes, and the activation of T cell bystander effects leading to B cell activation and antibody production [93,118].

**Table 2:** Biological pathways implicated in NCGS-related psychiatric symptoms.

Proposed Mechanism	Summary of Supporting Data	Comments/Limitations	References
**Gut dysbiosis**	Reduced microbial richness; ↑ *Ruminococcaceae*, ↓ *Bacteroidetes* and *Fusobacteria* in NCGS	Supported by human stool microbiome studies in NCGS patients; no prospective causal data available	[41,42,43]
**Tight junction disruption/zonulin (intestinal)**	Zonulin dysregulation increases intestinal permeability; elevated serum zonulin correlates with NCGS symptoms	Supported by human serum measurements and intestinal biopsy data; zonulin as a standalone diagnostic biomarker remains contested	[44]
**SCFA deficiency → barrier dysfunction**	Reduced SCFA production impairs epithelial integrity and T-reg cell generation	Supported by mechanistic human and rodent studies; direct SCFA quantification in NCGS patients is limited	[45,46,47,48,49,50]
**LPS/microbial translocation**	Elevated LPS-binding protein and intestinal FABP indicate compromised gut barrier and microbial translocation	Supported by cross-sectional human serum data in NCGS patients; causality not established	[25]
**Innate immune activation (TLR-2, IFN-γ, FOXP3, CXCL-10)**	Altered expression of innate immune markers in intestinal biopsies following gluten exposure	Directly supported by human intestinal biopsy and peripheral blood mononuclear cell studies	[24,32,33,34,35,36]
**Adaptive immune activation (AGAs, CD3+ T cells, CD45+ cells)**	~50% of NCGS patients show elevated anti-gliadin antibodies; elevated intraepithelial CD3+ T cells and lamina propria CD45+ cells	Supported by human serological and histological data; absence of anti-TG2 distinguishes NCGS extrapolated from CD	[37,38]
**Gut–brain axis dysregulation**	Bidirectional gut–brain communication via neural, endocrine, and immune pathways links gut pathology to psychiatric outcomes	Broadly supported across functional GI disorder and psychiatric neuroscience literature; NCGS-specific human theoretical data remain moderate in strength	[56,58,59,60,61]
**Gluten exorphins → BBB penetration → opioid system dysregulation**	Incomplete gluten digestion generates opioid-active peptides that may cross the BBB and alter neurotransmission	The hypothesis arises from leaky gut theory and peptide-detection studies, yet direct evidence that these peptides actually cross into the human central nervous system remains absent	[93,104,105]
**Zonulin → BBB disruption**	Overexpression of zonulin compromises blood–brain barrier permeability in much the same way it increases intestinal permeability.	Two independent human in vivo studies (CSF/serum albumin quotient; DCE-MRI) found zonulin plays a negligible role in BBB permeability; mechanism supported only by preclinical zonulin analogue experiments and should be presented as a contested hypothesis	[106,107,108]
**tTG6 exposure in neuronal cells**	Gluten peptides initiate innate immune responses in the brain via tTG6 from neuronal cells or macrophages	Supported by human serological data showing elevated anti-tTG6 antibodies in schizophrenia and gluten-related neurological disease; mechanistic pathway in neuronal cells remains preclinical and inferred by analogy with gut tTG2	[98,111,112,113]
**Serotonin/tryptophan depletion**	Wheat protein consumption reduces brain tryptophan availability, impairing 5-HT synthesis and contributing to depression	Wheat-specific tryptophan effect demonstrated in a single rodent study; the broader tryptophan–serotonin–depression axis is well-supported in human and animal literature, but a direct gluten-specific effect in humans has not been established	[65,70,71,72,73,74,75,76,77,78]
**Cortisol/HPA axis dysregulation**	Elevated cortisol associated with negative affect may link NCGS to mood disorders	No direct evidence for gluten-stimulated cortisol secretion; included for completeness as a theoretical pathway	[79,80,81]
**Gliadin-induced cytokine production (TNF-α, IL-12, IL-15, IFN-β)**	Gliadin activates a repertoire of pro-inflammatory genes in macrophages	Demonstrated in murine peritoneal macrophage cultures and supported by human monocyte studies; in vivo relevance in NCGS patients requires further confirmation	[114]

NCGS, non-celiac gluten sensitivity; SCFA, short-chain fatty acid; LPS, lipopolysaccharide; FABP/FABP2, (intestinal) fatty acid-binding protein/fatty acid-binding protein 2; TLR-2, Toll-like receptor 2; IFN-γ, interferon gamma; FOXP3, forkhead box protein P3; CXCL-10, C-X-C motif chemokine ligand 10; AGAs, anti-gliadin antibodies; CD, celiac disease; CD3+ T cells, CD3-positive T lymphocytes; CD45+ cells, CD45-positive leukocytes; BBB, blood–brain barrier; CSF, cerebrospinal fluid; DCE-MRI, dynamic contrast-enhanced magnetic resonance imaging; tTG6, tissue transglutaminase 6; tTG2, tissue transglutaminase 2; 5-HT, 5-hydroxytryptamine (serotonin); HPA axis, hypothalamic–pituitary–adrenal axis; TNF-α, tumor necrosis factor alpha; IL-12, interleukin 12; IL-15, interleukin 15; IFN-β, interferon beta; GI, gastrointestinal.

## 4. Psychiatric Manifestations in NCGS Patients

The typical presentation of NCGS includes a range of gastrointestinal symptoms such as abdominal pain, bloating, and irregular bowel habits, alongside extraintestinal manifestations encompassing psychiatric disorders such as depression, anxiety, psychosis and schizophrenia [54,55,57,104,119]. NCGS individuals are characterized by baseline differences in affect, higher acute fatigue and subacute gastrointestinal symptoms that may be explained by nocebo effects. Thus, NCGS appears to be a term encompassing individuals with varying combinations of disorders of gut–brain interaction, psychological distress, and food-related anxiety [120].

### 4.1. Depression

Symptoms of gluten-induced depression were noted in patients with NCGS, following brief exposure periods of just a few days [121]. In a cross-over trial of subjects with suspected NCGS, the severity of overall symptoms including depression increased significantly during one week of intake of small amounts of gluten, compared with placebo with one-week washout [122]. The fact that short-term gluten intake causes depression is striking, and this finding was corroborated by a double-blind, placebo-controlled trial in which depression was measured using an ad hoc psychiatric score. The results of an Australian clinical study indicated that gluten exacerbated depression scale scores in patients with a confirmed diagnosis of NCGS, whereas other symptoms, including anxiety, curiosity, and anger, were unaffected by the diet [65]. In the same study each dietary arm (gluten, whey, placebo) lasted only 3 days, with a 3–14-day washout between arms; state-depression was assessed on day 3 [65]. Although increased cortisol concentration is often associated with negative affective states including depression, Peters and coworkers found similar salivary cortisol levels between all dietary treatments. This study indicated that gluten ingestion in patients with NCGS may not directly induce depressive symptoms in a cortisol-dependent manner. Moreover, some authors hypothesized that gluten may induce depression as a consequence of changes in brain serotonin, gluten exorphins or changes in gut microbiota [65]. Bearing in mind that depression is a common occurrence in Western society; some authors suggest that it may represent a specific mood trait of personality rather than an extrinsic manifestation of NCGS. For example, individuals with higher neuroticism, who tend to be anxious, easily upset, moody, or depressed are sensitive to external stimuli including gluten and tend to report physical symptoms without relation to somatic illness [123]. However, although NCGS patients reported more abdominal and non-abdominal symptoms after gluten challenge than CD patients, these symptoms were not related to general somatization and personality in NCGS patients [124]. *Busby* et al. (2018) performed a systematic review with meta-analysis (13 studies, *n* = 1139) [125]. A long-term GFD significantly reduced and normalized severity of depressive symptoms (SMD −0.37, 95% CI −0.55 to −0.20, *p* < 0.0001), and after one year, GFD-treated patients showed no difference from healthy controls (SMD 0.01, *p* = 0.94). In two RCTs specific to NCGS, there was a trend toward worsened depression during gluten challenge vs. placebo (SMD 0.21, *p* = 0.25) [125]. In this systematic review improvements were in depressive symptoms tied to a long-term (≥1 year) GFD, after which treated patients no longer differed from healthy controls (SMD 0.01, 95% CI −0.18 to 0.20, *p* = 0.94) [125]. The two NCGS-specific RCTs contributing to this meta-analysis (SMD 0.21, 95% CI −0.58 to 0.15, *p* = 0.25) were precisely the short-term, blinded gluten-challenge trials by *Peters* et al. and *Di Sabatino* et al. Finally, it must be noted that the depressive changes demonstrated in NCGS to date derive almost entirely from challenge studies, lasting only days to one week, so they should be read as evidence of an acute, gluten-associated mood effect rather than of durable clinical benefit. Also, the evidence that a GFD produces sustained normalization of mood over months to a year comes largely from mixed populations (celiac disease, IBS and NCGS combined) rather than from NCGS alone.

Although the relationship between these outcomes remains theoretical, it can be proposed that a GFD may improve mental health outcomes in susceptible individuals by reducing inflammation as evidenced by the disappearance of anti-gliadin IgG antibodies in NCGS patients [126]. A fermentable oligo-, di-, monosaccharides and polyols (FODMAPs)-reduced diet can further alleviate depressive symptoms in NCGS patients already on a GFD [42], suggesting that NCGS may not always be an isolated condition and may involve interaction, potentiation, or overlap with other types of sensitivities. These psychological well-being effects probably involve gut microbiota shifts [42]; however, results should not be extrapolated from one population to another, due to the highly individualized pattern of gut microbial composition.

### 4.2. Anxiety Disorders

A recent study indicated that anxiety and depression are more common among patients with NCGS than in the general population [127]. *Iven* et al. demonstrated that individuals with NCGS exhibit distinct psychological characteristics at baseline, with higher negative affect and lower positive affect [120]. An Italian prospective multicenter survey on patients suspected of having NCGS demonstrated that the most frequent extraintestinal manifestations were tiredness and lack of well-being. In particular, a high prevalence of neuropsychiatric symptoms including anxiety (39%), ‘foggy mind’ (38%), and headache (54%) were recorded [119]. For this reason, from a clinical point of view, it is relatively difficult to differentiate NCGS from other conditions that share the same clinical features. As a result, in more than half of the instances, the patients self-diagnosed NCGS and began avoiding gluten in their diet on their own. This prospective study also revealed a substantial association between NCGS, female gender, and adult age [119]. Accordingly, in the recently published clinical study that analyzed the clinical/psychological characteristics of individuals with suspected NCGS, most of participants were females, around 40 years old, with normal body mass index (BMI) and a high level of education. NCGS individuals displayed an increased risk for eating disorders and a low mental health, and moreover, they all displayed anxiety, which was mostly moderate to severe [128]. Anxiety is frequently reported among NCGS patients, though specific RCT data is more limited: *Jackson* et al. (2012) found that gluten-related patients had significantly higher state anxiety than controls, with improvement after one year on a GFD [129]. The food-related anxiety and strong nocebo effects suggest that current diagnostic approaches may be insufficient and potentially symptom-reinforcing. Social phobia and panic disorder have also been associated with gluten response. A higher lifetime prevalence of panic disorder was found in CD patients, and these associations likely extend to NCGS patients who present predominantly with extraintestinal symptoms [129]. Despite the mentioned data, the latest study of *Schurdak* et al. (2025) [130] displayed opposing results; to investigate gluten’s impact on brain function, wild-type mice were fed a high-fat diet (35% kcal) with or without gluten. After 2-3 months, assessments of body weight, fat percentage, glucose tolerance, and behavioral tests were conducted with no significant differences in any of these parameters. Yet mice that received gluten showed reduced anxiety and increased locomotor activity in the elevated-platform maze, whereas performance in the Morris water maze and restraint stress test was similar across diets. This indicates that dietary gluten can affect behavior under high-fat-diet conditions. Important limitations must be considered: because no inflammation was detected, the study cannot determine whether the behavioral changes are related to central or peripheral inflammatory processes and any subtle gluten-related effects may have been masked by the overriding influence of the high-fat diet. Future studies directly examining these mechanisms are crucial to better understanding the anxiety in gluten-sensitive individuals.

### 4.3. Psychosis and Schizophrenia

Low-grade inflammation and increased intestinal permeability possibly initiated by processes in the gastrointestinal tract related to gluten hypersensitivity reactions may contribute to the pathophysiology of mental health manifestations including isolated psychosis and full-blown schizophrenia. *Dohan* explored the relationship between gluten and schizophrenia in a series of studies [19,20,21]. In his 1966 work, he observed that populations with lower grain consumption tended to have fewer cases of schizophrenia. He then showed that removing milk and cereals from the diet improved psychotic symptoms, allowing patients to be moved to an open ward more quickly than those who continued to eat gluten [19]. His later study found that patients adhering to the GFD were discharged at roughly twice the rate of those who did not follow the diet [20]. Conversely, another study reported that reintroducing gluten after a period of GFD disrupted the recovery that had been achieved [131]. In addition to these clinical observations, *Dohan* et al. injected rats intracerebrally with fractions of gliadin polypeptides [21]. At high doses, the animals exhibited seizures, perseverative (repetitive) behaviors, and other atypical responses that the authors described as reflective mechanisms involved in the pathogenesis of schizophrenia.

*Singh et al*. [131] conducted a controlled trial on a secure research ward with 14 participants who were first placed on a GFD and then given a gluten challenge. Blind raters noted significant improvement in 30 out of 39 outcomes covering psychopathology, social avoidance, and overall participation. A separate blinded study examined 16 individuals with chronic schizophrenia and reported observable changes in the Brief Psychiatric Rating Scale (BPRS) after a similar dietary manipulation [132]. Among these participants, two exhibited improvements in their functioning during the GF phase, while one of these two experienced significant regression during the subsequent gluten challenge [132]. *Vlissides* et al. conducted a double-blind trial of gluten-free versus a gluten-containing diet, and observed beneficial changes in five of the twelve metrics on the Psychotic In-Patient Profile (PIP) scale during gluten-free period [133]. Two patients relapsed when the gluten-containing diet was reintroduced, whereas the remaining patients maintained clinical improvements during the gluten challenge period, suggesting that the observed alteration could be attributed to the attention the patients received. However, it must be noted that some negative studies may have excluded patients with gluten sensitivity [134].

Afterwards, patients with recent-onset psychosis had elevated IgG and IgA antibodies to gliadin compared to controls, while multi-episode schizophrenia patients showed elevated IgG antibodies only [93]. *Cascella* et al. (2011) used blood samples from the CATIE schizophrenia trial (N = 1401) and found an anti-gliadin antibody (AGA) prevalence of 23.1% in schizophrenia patients versus 3.1% in the general population [94]. The increased AGA IgG (≥12 U/mL) antibody titer was associated with a four-fold higher ODDS of schizophrenia in patients with first-episode schizophrenia (OR = 4.00, 95% confidence interval (CI)): 1.43–11.19) and almost a seven-fold higher ODDS in the chronic schizophrenia patients (OR = 6.68, 95% CI: 1.71–25.04) [134]. Those identified in the gluten-sensitive enteropathy may differ from the epitopes recognized by AGAs detected in schizophrenia. In comparison to control subjects, patients with schizophrenia exhibited elevated plasma IgG levels against the indigestible γ-gliadin-derived fragment AAQ6C, but decreased plasma IgG levels against the α- and γ3-gliadin-derived antigens. Additionally, this investigation illustrated a consistent decline in plasma IgA antibodies directed at gliadin-derived antigens [135]. Importantly, in contrast to individuals with CD, the majority of NCGS patients with schizophrenia did not show elevated levels of anti-deamidated gliadin peptide (anti-DGP) and anti-tissue transglutaminase 2 (anti-TTG2) IgA [136]. Therefore, the development of schizophrenia in individuals with gluten-related disorders may be influenced by the interactions between the immune system and the central nervous system, which may result in injury from the antibodies to gluten or resultant immune-related mechanisms. [14]. Most recently, *Dzikowksi* et al. for the first time examined sex-related differences and illness stage in the immune response to gluten among patients with schizophrenia [137]. Stronger inflammatory responses, characterized by higher concentrations of AGA IgG and deamidated gliadin (dGP) IgG, were found in men, suggesting sex-related disparities in gluten-related immune activation [137]. These results underscore the intricate relationship between psychiatric symptoms, immune function, intestinal barrier integrity, and hormones.

*Genuis* et al. (2014) presented a case of NCGS manifesting as a longstanding neuropsychiatric disorder with auditory and visual hallucinations, achieving life-changing improvement through gluten elimination [55]. When re-exposed to gluten, relapse consistently occurred within 3–5 h and would result in significant disorientation and departure from reality. This gluten-related illness has been discussed in the contest of accumulated toxic insults resulting from gluten exposure led to hypersensitivity and impaired tolerance of the immune system (known as toxicant induced loss of tolerance or “TILT”). A later case report presented by *Lionetti* et al. displayed instances of “gluten psychosis” in a patient with NCGS [57]. In this patient, hallucinations, crying spells, significant confusion, ataxia, severe anxiety, and paranoid delirium manifested shortly after gluten ingestion. After the diagnosis of a fluctuating psychotic disorder, treatment with a second-generation anti-psychotic (i.e., olanzapine) was started in this 14-year-old girl, but psychotic symptoms persisted. Interestingly, after a nutritionist was consulted and GFD was recommended for symptomatic treatment of gastrointestinal symptoms and unexpected weight loss, and within one week of GFD, the symptoms (both gastrointestinal and psychiatric) dramatically improved [57]. Subsequently, *Levinta* et al. (2018) conducted a systematic review of nine studies on GFD in schizophrenia and found six of nine studies showed beneficial effects including improved functioning and decreased symptom severity [138]. These data suggested that GFD has minimal risk in patients with schizophrenia, and is a feasible option in terms of adherence. Recently, a case of a bipolar-type of schizoaffective disorder was reported in a 31-year-old woman with NCGS (no serological CD evidence), whose psychiatric symptoms improved with gluten elimination [60]. However, a dose-dependent response to gluten sensitivity, with prolonged rates of resolution following recurrent exposure, suggested that further studies are needed to clarify this potential dose-dependent association. In the same year, *Selmi* et al. (2023) described a 15-year-old girl with no psychiatric history who developed anxiety and paranoid delusions unresponsive to conventional treatment, but who significantly improved on a strict GFD [139]. By very strict GFD, she has achieved complete remission of psychotic symptoms and successfully returned to her pre-illness baseline in terms of social and academic functioning.

### 4.4. Symptom Exacerbation Versus De Novo Psychiatric Onset in NCGS

Taken together, the available literature points to two clinically distinct, though not always cleanly separable, patterns of psychiatric involvement in NCGS. On one hand, case reports describe patients who developed psychiatric symptoms—hallucinations, paranoid delusions, and confusional states—seemingly out of nowhere, with no prior psychiatric history, and who then recovered, often strikingly, once gluten was removed from their diet. *Lionetti* et al. offer a particularly illustrative example: a 14-year-old girl with no psychiatric background who developed hallucinations, paranoid delirium, and severe anxiety, with symptoms that resolved within just one week of gluten elimination [57], and *Selmi* et al. reported a comparable case of new-onset paranoid delusions in a 15-year-old with no psychiatric history, achieving complete remission on a strict GFD [139]. Second, a separate and larger body of evidence indicates that gluten exposure exacerbates pre-existing symptom severity in patients already carrying a psychiatric diagnosis, rather than inducing a distinct new disorder. This pattern is best documented for depression, where a systematic review and meta-analysis of GFD trials found that long-term adherence significantly reduced depressive symptom severity in patients with established depressive symptomatology, with a trend toward symptom worsening on gluten rechallenge in NCGS-specific randomized trials, and where short-term gluten exposure was shown to acutely worsen depression scores, without inducing new diagnostic entities, in patients with a confirmed diagnosis of NCGS [125]. Much the same pattern holds for schizophrenia: the older dietary intervention studies did not report gluten as a trigger for the disorder’s onset in previously healthy individuals, but rather documented symptom relapse and delayed clinical improvement when gluten was reintroduced into the diets of patients who already carried an established diagnosis [19,20,21,55,131,133]. Anxiety data follows a comparable exacerbation pattern, with gluten-related patients showing significantly elevated state anxiety relative to controls that improved after a year on a GFD. Most quantitative studies measure changes in symptom severity rather than applying formal diagnostic criteria, so we interpret the available evidence as predominantly reflecting a worsening of pre-existing psychiatric symptoms following gluten exposure. A smaller subset of case reports, however, tells a rather different story, one in which patients went on to develop entirely new psychotic or affective disorders in the context of NCGS.

## 5. Confounding Factors in NCGS-Related Psychiatric Symptoms

The studies reviewed herein span a wide methodological spectrum, from double-blind placebo-controlled trials and meta-analyses to observational studies and case reports. A substantial proportion of the evidence rests on limited sample sizes, and many investigations rely on self-reported gluten sensitivity rather than diagnosis confirmed by the double-blind placebo-controlled gluten challenge as defined by the Salerno criteria. The cross-sectional and observational nature of much of the available data precludes firm causal conclusions, and molecular pathways derived predominantly from animal or in vitro studies are noted as such throughout. The findings presented here should therefore be interpreted as converging, though not yet definitive, lines of evidence.

Interpreting the psychiatric symptoms associated with NCGS is rarely straightforward, and several confounding variables must be acknowledged before drawing firm conclusions from the available evidence. Wheat and related gluten-containing grains are simultaneously rich in fermentable oligosaccharides, disaccharides, monosaccharides, and polyols (FODMAPs) [140]. When FODMAP content is carefully matched between gluten and placebo arms in controlled rechallenge studies, the specific contribution of gluten to symptom provocation is substantially diminished—raising the genuine possibility that symptoms long attributed to gluten sensitivity may, in at least a subset of patients, reflect FODMAP intolerance rather than a response to solely gluten itself [63]. A consecutive double-blind, randomized, placebo-controlled, cross-over rechallenge study did not confirm specific or dose-dependent effects of gluten in patients with self-reported NCGS after a low-FODMAP diet [63]. On the contrary, a high nocebo effect and poor reproducibility of participants’ responses to gluten were observed, suggesting that FODMAPs may represent an important confounding factor in the interpretation of both gastrointestinal and psychiatric symptoms in NCSs and highlighting the need for rigorous diagnostic approaches. A non-trivial proportion of self-reported NCGS patients experience symptom worsening following gluten ingestion regardless of whether gluten was actually administered. Pooled analyses of double-blind placebo-controlled gluten challenge studies estimate that the nocebo response may account for symptom provocation in approximately 40% of this population [141]. Since mood-related outcomes such as anxiety and depression are particularly vulnerable to expectation-driven reporting, nocebo is an especially relevant confounder in studies relying on subjective psychiatric endpoints. In addition, unsupervised adoption of a GFD carries a well-documented risk of deficiencies in vitamin D, folate, iron, and B vitamins [142]—all of which have established roles in neurological and psychiatric function. Thus, improvements in mood or cognition observed following dietary change should therefore not be interpreted reflexively as evidence of gluten’s pathogenic role; they may equally reflect the correction of pre-existing nutritional deficits. Further, a substantial diagnostic overlap exists between NCGS and irritable bowel syndrome (IBS) [143], and the high prevalence of anxiety and depression in IBS is well-established [144]. This diagnostic overlap is further corroborated at the population level: a recent global meta-analysis reported a strong association between NCGWS and IBS (OR 4.78), a finding that lends credence to the concern that psychiatric symptoms attributed to NCGS may, at least in part, reflect the well-established psychiatric burden already associated with IBS itself [145]. Studies that do not formally exclude IBS, or control for its presence, risk attributing to gluten a psychiatric burden that more plausibly reflects the chronic functional disorder itself. This is in line with the latest study by *Iven* et al. (2025) [120]. These findings suggest that NCGS is marked by baseline differences in mood, along with heightened acute fatigue and sub-acute gastrointestinal discomfort that are not specifically tied to gluten. This pattern may reflect nocebo effects, indicating a need for further research into alternative mechanisms and a possible re-evaluation of how NCGS is defined [120]. Self-diagnosis poses another serious problem for the NCGS literature, largely because most epidemiological studies define their cases purely on the basis of symptom self-report rather than the more rigorous Salerno double-blind, placebo-controlled gluten challenge. A recent global meta-analysis, drawing on nearly 50,000 participants from 16 countries, put the prevalence of self-reported NCGWS at 10.3% (95% CI 7.0–14.0%), with women considerably more likely than men to report the condition (OR 2.29, 95% CI 1.80–2.90) [145]. The same analysis also found that self-reported NCGWS was significantly linked to both anxiety (OR 2.95) and depression (OR 2.42) at the population level, which lends some epidemiological weight to the clinical observations discussed earlier [145]. *Cha* et al. reached a similar conclusion using a rather different method: relying on a VAS symptom-severity threshold in place of an actual gluten challenge, they found self-reported NCGS in 5.8% of individuals without IBS, but in a striking 33.6% of those who met IBS criteria, a gap that speaks both to how unstable these self-report thresholds can be and to how heavily NCGS and IBS overlap in practice [146]. *Van Gils* et al. found a related, if somewhat different, observation: while 6.2% of a Dutch sample identified themselves as gluten-sensitive, fewer than one in five ever brought the issue to a doctor, and only 4% went so far as to adopt a strict GFD, suggesting that most self-reported cases are never clinically confirmed and are, at best, only half-heartedly acted upon [147]. Much the same pattern turns up in more specific patient groups: *Seidita* et al., studying a cohort of patients with Sjögren’s syndrome, reported self-identified NCWS in nearly half of participants, but were careful to note that because these findings rested entirely on questionnaire responses rather than gold-standard challenge testing, they ought to be interpreted with real caution [148]. Taken together, these studies suggest that self-diagnosis tends to inflate apparent prevalence, draws disproportionately on individuals who already have functional or psychiatric symptoms, and offers no real assurance that what is being captured is a genuine, biologically confirmed response to gluten. None of these considerations invalidate the existing evidence for NCGS-associated psychiatric symptoms. They do, however, make a compelling case for more rigorous study designs.

## 6. Conclusions

NCGS occupies an uncomfortable position in clinical medicine—real enough to cause genuine discomfort, yet poorly defined enough to remain on the margins of mainstream diagnostic practice. This review has attempted to bring greater clarity to one of its most overlooked dimensions: the psychiatric burden that a meaningful proportion of affected patients carry, often without ever receiving an explanation for their symptoms. The evidence, taken together, paints a coherent if still incomplete picture. Disruption of the intestinal barrier, shifts in gut microbial composition, and the downstream activation of neuroimmune pathways create conditions in which gluten exposure may plausibly contribute to anxiety, mood disturbances, and psychotic episodes. None of these links are yet definitive, and the methodological limitations of existing studies—small samples, self-reported diagnoses, and insufficient blinding—demand that conclusions be drawn with appropriate restraint. Although current evidence remains limited, the available literature suggests that NCGS patients’ psychiatric symptoms should not be dismissed as functional or psychosomatic without appropriate clinical evaluation. While many individuals with self-reported NCGS report symptoms exhibit improvement when gluten is removed from their diet, evidence from double-blind placebo-controlled gluten challenge studies suggests that only a minority represent true gluten-responsive cases, however it remains unclear why this happens and which patients are most likely to respond. Answering these questions should be a priority for future research—one that will depend on developing more reliable diagnostic tools, conducting larger and better-designed clinical trials, and encouraging clinicians from different specialties to collaborate when patients present with complex, overlapping symptoms that do not fit neatly into a single disease category.

## Figures and Tables

**Table 1:** Overview of the Salerno criteria for diagnosing NCGS.

Component	Description
**Symptom assessment tool**	Self-administered instrument based on a modified Gastrointestinal Symptom Rating Scale; patient selects 1–3 main symptoms
**Symptom scoring**	Numerical Rating Scale, range 1–10, applied to each of the selected symptoms
**GFD run-in**	Baseline gluten-containing diet for ≥6 weeks, followed by GFD with weekly symptom monitoring for 6 weeks (Step 1); strict GFD for ≥4 weeks required before gluten challenge (Step 2)
**Gluten challenge design**	Double-blind, placebo-controlled, cross-over design; 8 g/day cooked, homogeneously distributed gluten vehicle
**Challenge duration**	One-week challenge, one-week washout on strict GFD, then cross-over to the second one-week arm (gluten or placebo)
**Positivity threshold**	≥30% variation in nominated symptom score between gluten and placebo arms required to call the result positive

Data derived from *Catassi* et al., 2015 [5].

## Data Availability

No new data were created or analyzed in this study. Data sharing is not applicable to this article.

## Abbreviations

The following abbreviations are used in this manuscript:

5-HT	5-hydroxytryptamine (serotonin)
AGAs	anti-gliadin antibodies
BBB	blood–brain barrier
CD	celiac disease
CNS	central nervous system
CSF	cerebrospinal fluid
CXCL-10	C-X-C motif chemokine ligand 10
DCE-MRI	dynamic contrast-enhanced magnetic resonance imaging
DPP-IV	dipeptidyl peptidase IV
FABP/FABP2	fatty acid-binding protein/fatty acid-binding protein 2
FODMAPs	fermentable oligo-, di-, monosaccharides and polyols
FOXP3	forkhead box protein P3
GFD	gluten-free diet
GI	gastrointestinal
GRDs	gluten-related disorders
HPA axis	hypothalamic–pituitary–adrenal axis
IBS	irritable bowel syndrome
IFN-β	interferon beta
IFN-γ	interferon gamma
IL	interleukin
iNOS	inducible nitric oxide synthase
LPS	lipopolysaccharide
MHC II	major histocompatibility complex II
NCGS	non-celiac gluten sensitivity
PIP	Psychotic in-patient profile
SCFA(s)	short-chain fatty acid(s)
SP	substance P
TLR-2	Toll-like receptor 2
TLR4	Toll-like receptor 4
TNF-α	tumor necrosis factor alpha
tTG2	tissue transglutaminase 2
tTG6	tissue transglutaminase 6
WA	wheat allergy
